# Supercritical Fluid Microcellular Foaming of High-Hardness TPU via a Pressure-Quenching Process: Restricted Foam Expansion Controlled by Matrix Modulus and Thermal Degradation

**DOI:** 10.3390/molecules27248911

**Published:** 2022-12-15

**Authors:** Bichi Chen, Junjie Jiang, Yaozong Li, Mengnan Zhou, Zelin Wang, Liang Wang, Wentao Zhai

**Affiliations:** 1 School of Materials Science and Engineering, Sun Yat-sen University, Guangzhou 510275, China; 2 Ningbo Key Lab of Polymer Materials, Ningbo Institute of Materials Technology and Engineering, Chinese Academy of Sciences, Ningbo 315201, China; 3 University of Chinese Academy of Sciences, Beijing 100049, China

**Keywords:** high-hardness, thermoplastic polyurethane, pressure-quenching foaming, matrix modulus, thermal degradation

## Abstract

High-hardness thermoplastic polyurethane (HD-TPU) presents a high matrix modulus, low-temperature durability, and remarkable abrasion resistance, and has been used in many advanced applications. However, the fabrication of microcellular HD-TPU foam is rarely reported in the literature. In this study, the foaming behavior of HD-TPU with a hardness of 75D was investigated via a pressure-quenching foaming process using CO_2_ as a blowing agent. Microcellular HD-TPU foam with a maximum expansion ratio of 3.9-fold, a cell size of 25.9 μm, and cell density of 7.8 × 10^8^ cells/cm^3^ was prepared, where a high optimum foaming temperature of about 170 °C had to be applied with the aim of softening the polymer’s matrix modulus. However, the foaming behavior of HD-TPU deteriorated when the foaming temperature further increased to 180 °C, characterized by the presence of coalesced cells, microcracks, and a high foam density of 1.0 g/cm^3^ even though the crystal domains still existed within the matrix. The cell morphology evolution of HD-TPU foam was investigated by adjusting the saturation time, and an obvious degradation occurred during the high-temperature saturation process. A cell growth mechanism of HD-TPU foams in degradation environments was proposed to explain this phenomenon based on the gas escape through the defective matrix.

## 1. Introduction

Thermoplastic polyurethane (TPU) is synthesized from polyether/polyester polyols, diisocyanatos, and chain extenders, in which polyols act as soft segments (SSs) and isocyanates and chain extenders serve as hard segments (HSs) [1]. TPU has a special microphase separation structure as a result of the incompatibility between HSs and SSs [2,3,4]. Due to its unique structures, TPU exhibits remarkable abrasion resistance, low-temperature durability, and high mechanical strength [5,6]. Compared with regular TPU, TPU with a hardness higher than shore 50D or shore 95A (HD-TPU) presents a higher matrix modulus, better abrasion resistance, and superior thermal stability [7,8], which broaden TPU’s usage in chemical mechanical polishing and other advanced applications in engineering fields [9,10].

The physical foaming behavior of TPU has been widely investigated by researchers [11,12], where different foaming techniques, such as autoclave bead foaming [13,14,15], compression molding foaming [16], injection molding foaming [17], have been used. Microcellular TPU foams with various shapes, such as bead foam [14], foam board [16], and foam film [18], and various cell sizes from nano-sized to micro-sized [6,19,20], have been fabricated. The motivations behind this research are that microcellular TPU foams possess soft touching, excellent elastomeric properties, and superior ductility, and are also 100% melt recycled, which has promoted the application of TPU foams in shoes, cushioning, sports, etc. [9]. Even though much progress has been made in research and application, the regular TPU foam is suffering from a low expansion ratio, with values usually less than 10 times, resulting from the serious post-shrinkage mainly due to its low matrix modulus [11].

HD-TPU presents high matrix modulus, high melting temperature (*T*_m_), and a rapid crystallization rate during melt-cooling. The rigid nature of HD-TPU could resist the post-shrinkage of the initial foam occurring in the early stage of cell structure stabilization and provide an opportunity to prepare HD-TPU foam with a higher expansion ratio. Unfortunately, the physical foaming of HD-TPU is rarely reported. In a recently published paper [21], Jiang et al. investigated the foaming behaviors of three HD-TPU foams using a temperature-rising foaming method. Various parameters, i.e., saturation pressure, foaming temperature, and foaming time, were carried out to adjust the foam expansion ratio and cell structure, and a maximum expansion ratio of about 10 times was obtained for 75D-HD-TPU foam using a mild saturation (6 MPa/25 °C/24 h) and foaming (170 °C/30 s) condition.

The physical foaming processes, which include temperature-rising method and pressure-quenching method, are the green way to produce polymeric foams [22]. Compared with temperature-rising foaming, pressure-quenching foaming contains a high-temperature and a high-pressure gas saturation process. A rapid gas diffusivity at high temperature shortens saturation time and enhances processing efficiency, which is more popular in industrial foam processing. In addition, a high-temperature treatment is helpful for generating favorable crystal domains [23,24,25,26]. In the case of HD-TPU, the presence of crystals can act as a nucleating agent to enhance heterogeneous nucleation [11], and the favorable crystal domains can work as a crosslinking point to increase the “melt” strength. Both contributions of crystal domains facilitate the cell growth and foam expansion of HD-TPU. Therefore, the pressure-quenching foaming method could be an effective strategy to obtain HD-TPU foams with an increased expansion ratio.

In this study, HD-TPU with hardness 75D was selected, and a pressure-quenching foaming method was applied to fabricate microcellular foams. The ordered microstructure evolutions of HD-TPU during the high-temperature annealing process were investigated by differential scanning calorimetry (DSC) and Fourier transform infrared spectroscopy (FTIR). Dynamic mechanical analysis (DMA) was carried out to explore the changes in matrix modulus as a function of temperature. A series of pressure-quenching foaming experiments with varied saturation temperatures and saturation times were conducted to prepare HD-TPU foams, and their effects on foam expansion ratio and cell structure were discussed. The change in the molecular weight of HD-TPU before and after foaming at different saturation times was investigated, and a possible cell growth mechanism at the degradation environment was proposed.

## 2. Results and Discussion

### 2.1. Ordered Microstructure Evolution of HD-TPU

The pressure-quenching foaming method involves an isothermal saturation process of compressed fluid, where the ordered microstructures tend to develop with the extension of saturation time, which influences cell nucleation and thus the cell morphologies of foams. HD-TPU exhibits increased crystallization ability compared with regular TPU resins with lower hardness. Here, the ordered microstructure evolution of HD-TPU is investigated first.

#### 2.1.1. Isothermal Annealing Analysis

**Figure 1:**
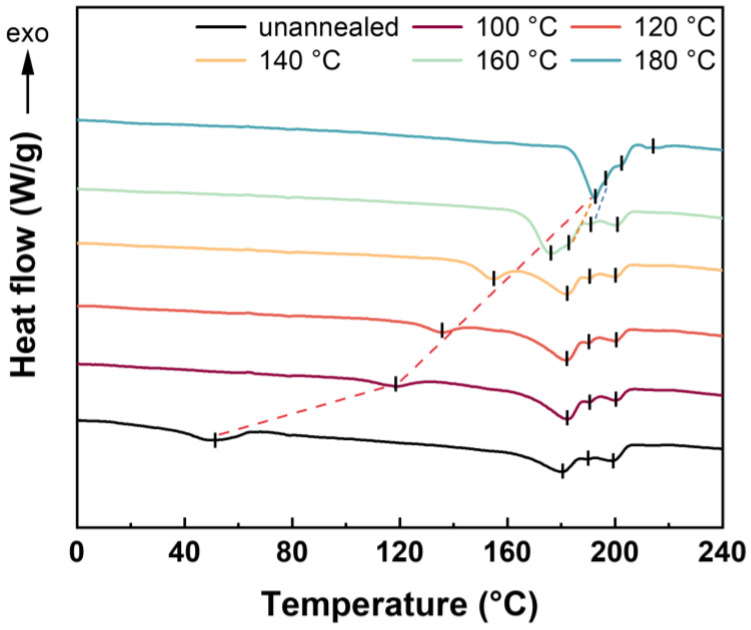
DSC heating curves of unannealed HD-TPU sample and samples after annealing at different temperatures.

Figure 1 presents the DSC thermograms of unannealed HD-TPU and samples annealed at different temperatures and atmospheric pressures. Four endothermic peaks could be identified in the DSC curves of the unannealed sample, namely *T*_m1_, *T*_m2_, *T*_m3_, and *T*_m4_, respectively, from low temperature to high temperature. This kind of multiple endothermic behavior was related to HSs domains with various order degrees [23,27,28,29]. Table 1 shows the specific data of these endothermic peaks. As seen in Table 1, the *T*_m1_ of the unannealed HD-TPU sample was 51.2 °C, which resulted from the cooling process during film extrusion. With the introduction of the annealing treatment, *T*_m1_ tended to increase dramatically up to 118.4 °C at the annealing temperature of 100 °C. At higher annealing temperatures, *T*_m1_ increased linearly with an increase in annealing temperature. The *T*_m2_, *T*_m3_, and *T*_m4_ of unannealed HD-TPU were about 180.4, 190.0, and 199.3 °C, respectively. They did not show any obvious changes during annealing treatments of 100–160 °C, indicating that the ordered microstructures of HD-TPU were perfect and thermally stable. This phenomenon is quite different from that of TPU with low hardness. For example, Jiang et al. found that the multiple *T*_ms_ of 85A-TPU all increased with the increase in annealing temperatures [30].

As the annealing temperatures further increased up to 180 °C, all the four *T*_ms_ increased up to 192.5, 194.4, 201.8, and 211.6 °C, respectively. This means that the different order degrees of HSs domains would go through processes of dissociation (or melting) and reaggregation (or recrystallization) [31,32], which were accompanied with improvements in long-range-ordered HSs domains.

#### 2.1.2. Microphase Separation Behavior

**Figure 2:**
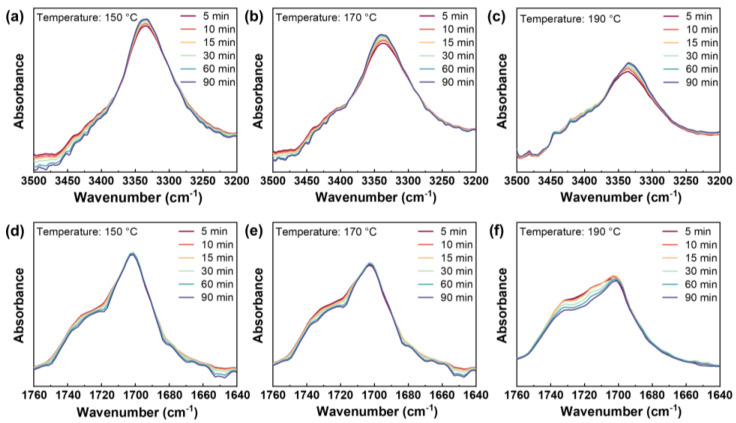
N-H peak spectra over time during annealing at (**a**) 150 °C, (**b**) 170 °C, and (**c**) 190 °C. C=O peak spectra over time during annealing at (**d**) 150 °C, (**e**) 170 °C, and (**f**) 190 °C.

Figure 2 shows the time scanning FTIR spectra of HD-TPU upon heating at 150–190 °C in the region of 3500–3200 cm^−1^ and 1760–1640 cm^−1^. The peaks at 3430 cm^−1^ and 3332 cm^−1^ were assigned to the N-H stretching of “free” and hydrogen-bonded N-H groups, respectively [4,33,34], while the peaks at 1730 cm^−1^ and 1700 cm^−1^ were assigned to the C=O stretching of “free” and hydrogen-bonded C=O groups, respectively.

As seen in Figure 2, the absorption peak intensity of the free groups gradually declined with the extension of heating time. The weakening of the free N-H peak indicated the reduction in free HSs, while the decrease in free C=O suggested that more HSs participated in the construction of the HSs domains [34]. For samples annealed at 150 °C and 170 °C, the intensity of the hydrogen-bonded N-H peak increased with time, while the intensity of the hydrogen-bonded C=O peak is almost unchanged. This implied that the possible formation of HSs domains with a higher-order degree arose from the rearrangement of HSs. For the latter, due to the dissociation of HSs and the reborn hydrogen bonds during the rearrangement process, the degree of hydrogen-bond association would essentially remain constant. In general, this process not only increased the hydrogen bonds between the HSs and SSs but also resulted in the formation of the higher-ordered HSs domains. When the temperature was increased to 190 °C, the intensities of the free C=O peak and the hydrogen-bonded C=O peak weakened over time, which was associated with the reduction in HSs domains through the dissociation of hydrogen bonds. The bonded N-H peak’s intensity continued to increase, indicating that the hydrogen bonds between HSs and SSs kept developing. This demonstrated that the microcrystalline HSs had been considerably melted at 190 °C, which exactly corresponded to the thermal behavior of *T*_m3_ in the DSC results of the unannealed sample.

### 2.2. Dynamic Mechanical Analysis

**Figure 3:**
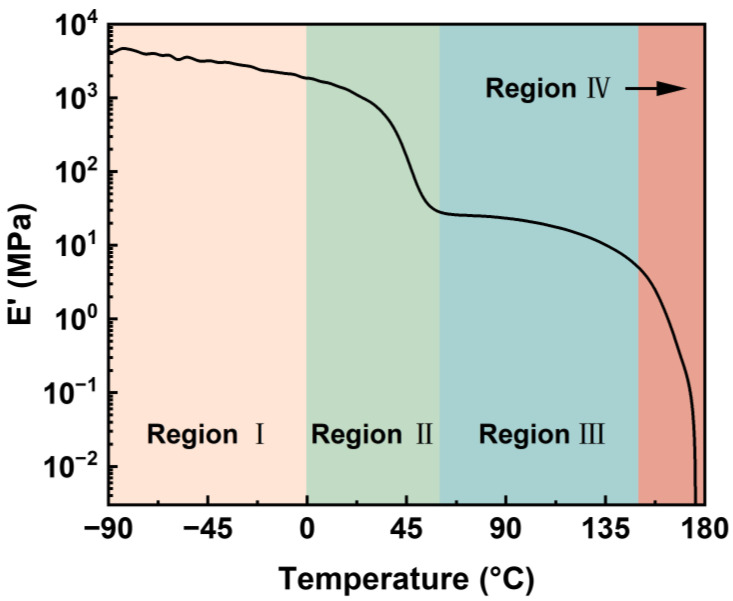
Storage modulus of HD-TPU as a function of temperature.

Figure 3 shows the storage modulus (E’) of HD-TPU as a function of temperature ranging from −90 °C to 180 °C. The modulus curve could be roughly divided into four regions: below 0 °C, 0–60 °C, 60–150 °C, and above 150 °C. When the temperature was below 0 °C, the decrease in the modulus was mainly due to the secondary relaxation and the motion of SSs. When the temperature was over *T*_g_ of the SSs, the SSs entered the rubbery state, while the HSs were still in the glassy state [21,35]. The increase in the SS’s mobility and the expansion of the free volume resulted in a slight drop in the storage modulus. Within 0–60 °C, the increase in temperature reduces the storage modulus by two orders of magnitude. The sharp decline in modulus at 49 °C in Region II could be ascribed to the *T*_g_ of HSs in HD-TPU. Within 60–150 °C, the ordered HSs domains created by the aggregation of HSs served to maintain the modulus in this range [36]. The slight drop in modulus could be related to the reorganization of ordered HSs into bigger HSs microdomains [37]. When the temperature was above 150 °C, the storage modulus of HD-TPU dramatically fell as a result of the melting and dissociation of HSs microdomains. The sharp decline in modulus was close to the DSC result, where the onset melting temperature of the unannealed sample was around 160 °C. The difference between the two temperatures is due to the distinct heating rates during measurements.

### 2.3. Foaming Behaviors of HD-TPU Sheets

#### 2.3.1. Effect of Saturation Temperature

**Figure 4:**
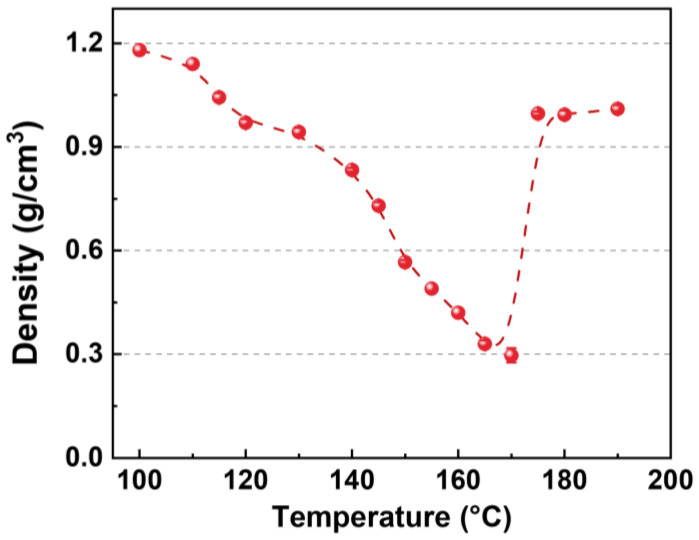
Density of HD-TPU foams as a function of saturation temperature. The saturation pressure and time were 18 MPa and 30 min, respectively.

The influence of saturation temperature on the foaming behavior of HD-TPU was investigated, where the saturation pressure was 18 MPa and the saturation time was fixed for 30 min. Figure 4 shows the density of HD-TPU foams as a function of saturation temperature. It is seen that no density reduction was observed when the saturation temperature was 100 °C. With the increase in saturation temperature, the foam density continuously declined and reached a lowest value of 0.31 g/cm^3^ at 170 °C. It was noteworthy that the density sharply climbed to 0.9 g/cm^3^ when the saturation temperature reached 175 °C. Similar considerable increases in foam density within short temperature ranges were also reported by Jiang et al. [38] in their work on the autoclave foaming of thermoplastic polyamide elastomer.

**Figure 5:**
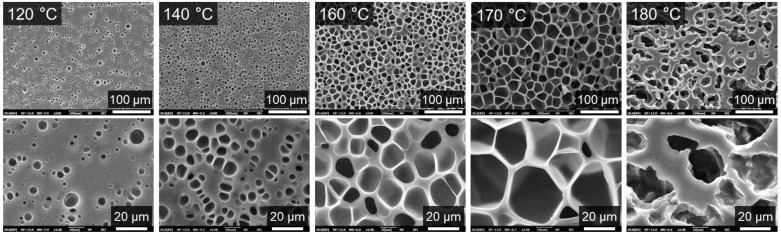
SEM images of cell morphology in HD-TPU foams prepared at different saturation temperatures. The bottom row of the image is a partial enlargement of the top row.

**Figure 6:**
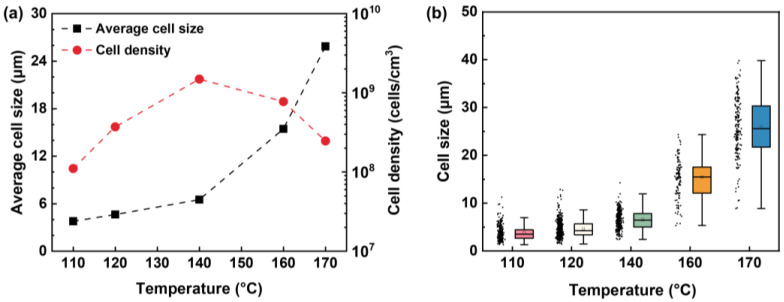
(**a**) Average cell size and cell density of HD-TPU foams as a function of saturation temperature. (**b**) Cell size distribution of HD-TPU foams as a function of saturation temperature.

Figure 5 shows the cell morphologies of HD-TPU foams obtained at various foaming temperatures, and Figure 6 summarizes the cell size, cell size distribution, and cell density of foams. At low foaming temperatures of 120 and 140 °C, the average cell size of foams is 3.7 and 4.6 μm, respectively, and the cell structures are circular and elliptical with broad cell size distribution randomly dispersed within the polymer matrix. These phenomena were attributed to crystal-domains-induced heterogeneous cell nucleation [39]. DSC analysis indicated that HD-TPU possessed multiple crystal domains with various degrees of order; therefore, the nonuniform crystal size and distribution were prone to induce nonuniform cell nucleation. These nonuniform cell nucleation phenomena were directly reflected in a SEM micrograph since the high matrix modulus at low foaming temperatures restricted the growth of nucleated bubbles.

As the foaming temperature increased to 160 °C, the cell size increased dramatically up to 15.5 μm and cell density decreased slightly from 1.5 × 10^9^ cells/cm^3^ at 140 °C to 7.7 × 10^8^ cells/cm^3^, and the cell structure became elliptical and uniform, characterized by the narrowed cell size distribution. The increase in foaming temperature could bring two contributions. One is that the decrease in matrix modulus was conducive to cell growth and foam expansion, and the other is that an increase in order degree and the homogenization of crystal HSs domains were helpful in inducing more uniform cell nucleation. A cell collapsing phenomenon usually occurs at the early stage of cell growth [40], where the big-size nucleated bubbles can “eat” nearby small bubbles, which will reduce the amount of small bubbles. Therefore, at the foaming temperature of 160 °C, HD-TPU exhibited a low matrix modulus and improved crystal features, all the effects of the homogenized heterogeneous cell nucleation induced by crystal domains, cell collapsing, and fully cell growth are helpful for the formation of increased cell size and improved cell size distribution. By further increasing the foaming temperature to 170 °C, the cell structure became polygonal, with a cell size of 25.9 μm and cell density of 7.8 × 10^8^ cells/cm^3^, and the foam density reached its maximum value of 3.9-fold. A further-decreased matrix modulus facilitated cell growth and foam expansion, while the decreased number of crystal domains tended to reduce the heterogenous nucleation effect, resulting in decreased cell density.

At the foaming temperature of 180 °C, HD-TPU foam presented coalesced cell structure and even open cells in some regions, which were accompanied by a sharp increase in foam density from 0.31 to 1.0 g/cm^3^ at 170 °C. This phenomenon is called cell coarsening [41], where cell structures are broken by high-pressure fluids because of the dramatically reduced “melt” strength of the polymer matrix. The reduced matrix modulus was observed in the DMA test, as indicated in Figure 3. One possible reason for it was that there were more HSs domains melted, as shown in DSC and FTIR tests. However, our research observed that the obvious chain degradation occurred at the foaming temperatures of 175–190 °C, which could be a major factor affecting cell growth; therefore, this issue and its possible mechanism will be discussed later.

**Figure 7:**
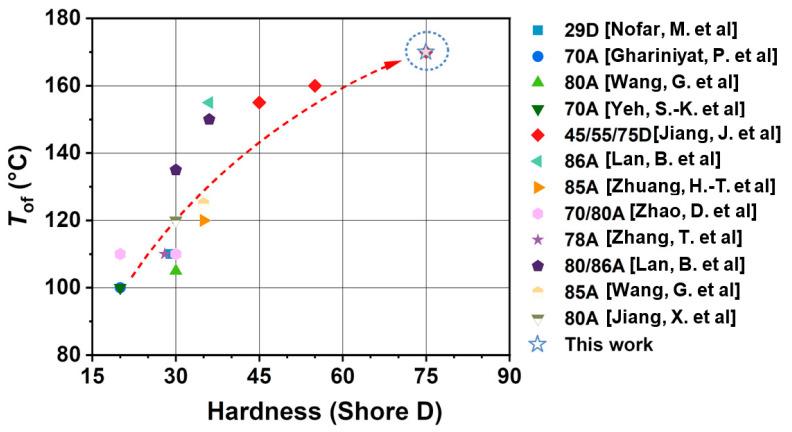
The relationship between hardness of resin and *T*_of_ [6,16,21,42,43,44,45,46,47,48,49,50].

As mentioned above, the foaming temperature is a critical factor to control foam expansion, and a mountain-shaped relationship between foaming temperature and foam expansion ratio has been widely reported [38]. Figure 7 summarizes the optimum foaming temperature (*T*_of_) of TPU as a function of sample hardness [6,16,21,42,43,44,45,46,47,48,49,50], where the compressed CO_2_ was used as the blowing agent. It is seen that an increase in resin hardness tends to increase the *T*_of_ of TPU foams. For TPU resins, a high hardness usually means a high matrix modulus, high crystallization ability, and high *T*_m_. In the case of HD-TPU, the increased foaming temperature was required to soften the polymer matrix for achieving the full growth of nucleated bubbles during the foaming.

#### 2.3.2. Effect of Saturation Time

**Figure 8:**
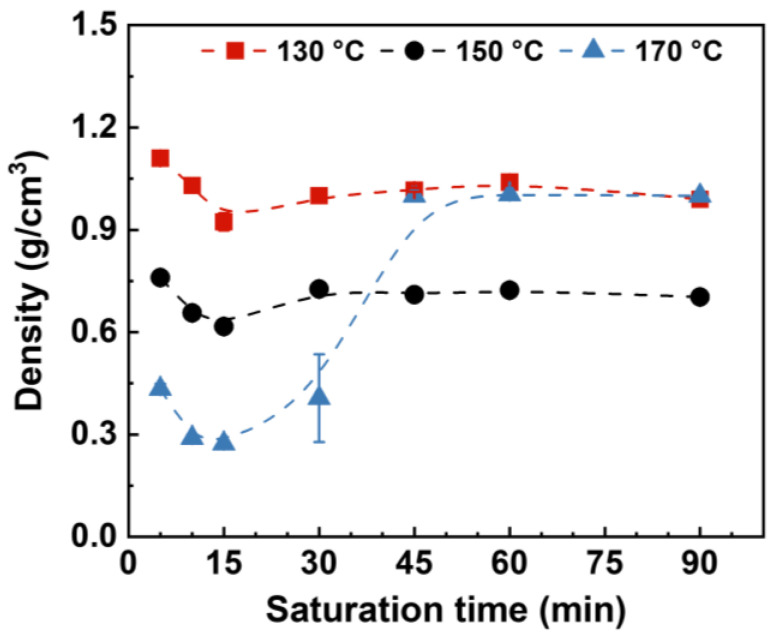
Density of HD-TPU foams as the function of saturation time. The saturation pressure was 15 MPa.

**Figure 9:**
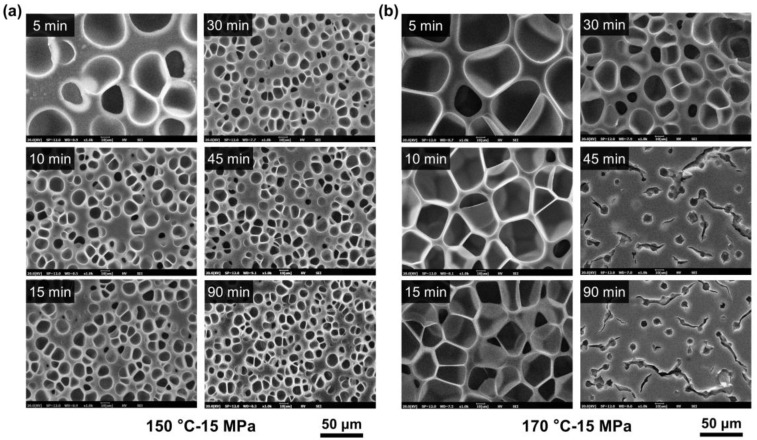
Cell morphology evolution of HD-TPU foams prepared at (**a**) 15 MPa/150 °C and (**b**) 15 MPa/170 °C for different saturation times.

During the saturation process, supercritical fluid dissolves in a polymer matrix and eventually reaches absorption equilibrium. Meanwhile, this saturation process can induce crystallization and thus affect the foaming behavior of semicrystalline polymers [26]. Figure 8 plots the density of HD-TPU foams saturated at 15 MPa/150–170 °C for various times, and Figure 9 shows the cell morphologies of the foams. The shortest saturation time of 5 min was selected since the compressed fluid presented high gas diffusivity, and the longest saturation time of 90 min was used to ensure the achievement of gas absorption equilibrium. It is seen that the densities of HD-TPU foams obtained at 130 and 150 °C were reduced a little bit by extending the saturation time from 5 to 15 min, and then tended to level off at longer saturation times. The cell morphologies shown in Figure 9 indicate that HD-TPU foam obtained at 5 min had larger cell size, and the cell size tends to decrease and then level off when the saturation time is longer than 15 min. These results demonstrates that the saturation time of 15 min was enough for reaching a gas dissolution equilibrium at 15 MPa/150 °C. However, the crystal domain evolution with the extension of saturation time seems too small to obviously affect the cell morphologies and foam expansion. Similar phenomena occurred at 15 MPa/130 °C.

As shown in Figure 8, HD-TPU presents a very different foaming behavior at the saturation condition of 15 MPa/170 °C, where the extension of saturation time from 5 to 15 min facilitated density reduction and cell size reduction, while a further increase in saturation time significantly increased foam density and spoiled the cell morphology of the foams. The cell morphologies of HD-TPU foam obtained at 15 MPa/170 °C/45–90 min were quite similar to that obtained at 18 MPa/180 °C/30 min; however, the former possessed a circular cell size and some microcracks through these cell structures. This phenomenon implies that the serious cell coalescence had been occurred during the foaming, and the gas escape and polymer chain relaxation led to the formation of microcracks and shrinkage of cell structure.

**Figure 10:**
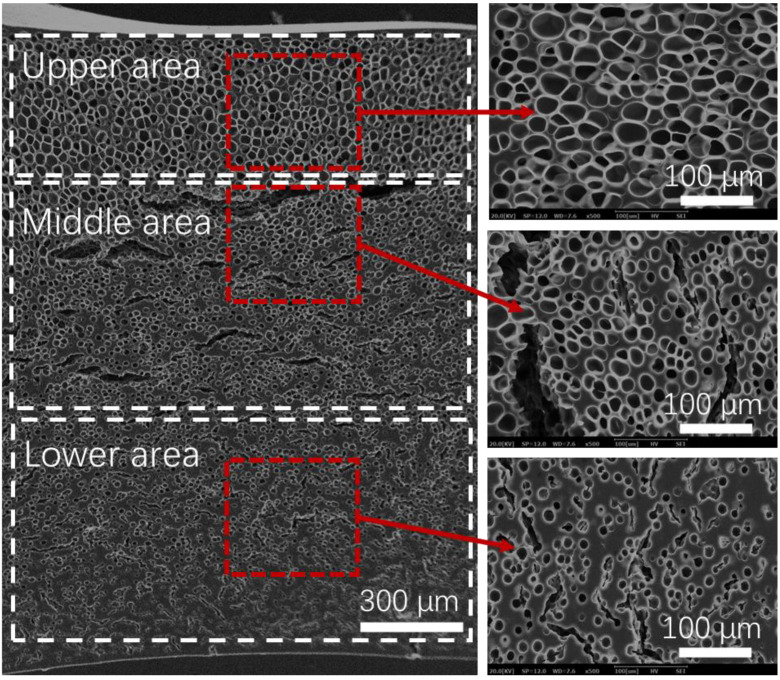
Cell morphologies of HD-TPU foam across the thickness direction. The foam was prepared at 15 MPa/170 °C/30 min.

During the saturation process, the HD-TPU sample was placed horizontally in the mold cavity, with one side of sample (upper area) contacting the metal mold and the other side (lower area) not. A further SEM observation was carried out for HD-TPU foam obtained at 15 MPa/170 °C/30 min to detect cell structures across the thickness direction of the foams. As shown in Figure 10, the upper area of HD-TPU foam exhibited a polygonal cell structure and uniform cell distribution, while the lower area presented lots of large-sized cracks, microcracks, coarsened cells, and circular cells. These coarsened cell morphologies were the same as those observed at 18 MPa/180 °C/30 min, and the circular cell morphologies were the same as those observed at 15 MPa/170 °C/45–90 min. This demonstrated that the cell coalescence phenomenon occurred at the lower area, while the normal cell growth was still in progress at the upper area. This kind of foaming behavior was very rarely reported by other researchers. When the foam sample was clearly examined, we did not observe any obvious melting trace on either side of the sample, and the lower area was more brittle and rigid than the upper area.

**Figure 11:**
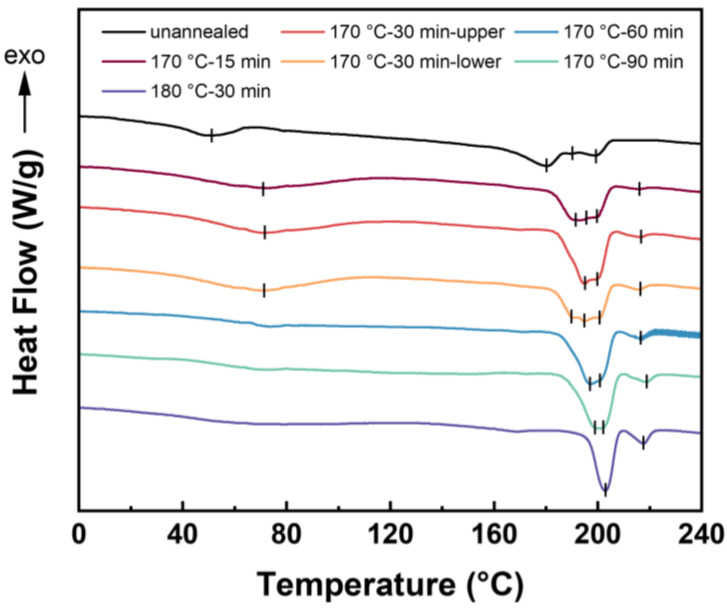
DSC curves of HD-TPU foams prepared at various saturation times. The saturation temperatures were 170 and 180 °C and saturation pressure was 15 MPa.

DSC analysis was carried out to investigate the crystal melting behaviors of HD-TPU before and after foaming, and the results are shown in Figure 11. It is observed the multiple *T*_mx_ around 200 °C was present before and after the foaming at 15 MPa/170–180 °C, and they only slightly increased; additionally, their width was narrowed with the extension in saturation time and increase in saturation temperature. These results demonstrated that the crystal domains corresponding to the *T*_mx_ around 200 °C still existed during the foaming at 170 and 180 °C. These crystal domains could act as physical crosslinking points to strengthen the “melt” strength and to then resist the cell coalescence during the foaming of HD-TPU. This generally accepted hypothesis seemed to be inconsistent with the cell morphologies results in Figure 5, Figure 10, and Figure 11, which indicated that there were other mechanisms determining cell growth behavior at a high saturation temperature or long saturation time.

### 2.4. Molecular Weight Change of HD-TPU before and after Foaming

Figure S1 (Supplementary Material) shows the retention time of unfoamed HD-TPU sheets and foams prepared at different conditions during the GPC test. The relative molecular weight (RMW) results are listed in Table 2. For the unfoamed HD-TPU sheets, the *M*_w_ is 181.5 kg/mol and the polydispersity index (PDI) is 1.8. The high-temperature gas saturation at 15 MPa/170 °C seems to gradually decrease the *M*_w_ of HD-TPU foams, which was 178.0, 158.6, 121.2, and 120.7 kg/mol at saturations of 5, 15, 60, and 90 min, respectively. Furthermore, both sides of HD-TPU obtained at 30 min presented different *M*_w_, where the upper area was about 144.7 kg/mol while the lower was about 133.5 kg/mol. These results demonstrated that the high-temperature treatment reduced the *M*_w_, and once the *M*_w_ was lower than the specific value, the chain degradation tended to affect cell growth dramatically, leading to the generation of coarsened cell structures.

**Figure 12:**
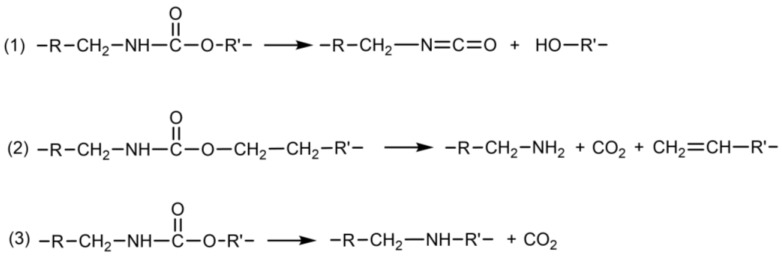
Three possible degradation pathways of TPU under high-temperature treatment.

It is well known that the degradation of TPU was usually divided into two stages. The first stage is the depolymerization of the urethane link and the second stage is the degradation of the macrodiol [51]. Additionally, usually, there were three main pathways for the initial degradation of TPU, which are shown in Figure 12. In particular, many scientists are convinced that Reaction 1 is the major degradation route for the degradation of the urethane link in TPU in the inert atmosphere [52,53,54]. Meanwhile, Hwang et al. [55] reported that the thermal degradation of TPU had taken place above 180 °C for more than 5 min. Yang et al. [55] studied the thermal degradation of TPU with HS formed of MDI/BDO using DSC and GPC. They found that above 170 °C, whether in a crystalline or molten state, polyurethane bonds became unstable. Therefore, the reduction in the *M*_w_ of HD-TPU foams at 170 °C mentioned in this study mainly belonged to the depolymerization of the urethane link. Moreover, when the break of the urethane link took place, the chain with large *M*_w_ transformed into some chains with small *M*_w_ and even small molecules, leading to changes in hard domains or crystallization.

### 2.5. The Cell Growth Mechanism of HD-TPU Foams in Degradation Environment

Increasing the saturation/foaming temperature is a general method to prepare polymeric foams with a higher expansion ratio in the pressure-quenching foaming process [56]. An increase in the temperature reduces the modulus and viscosity of the polymer, which contributes to the sufficient growth of nucleated cells, thereby increasing the expansion ratio of the foams. The expansion ratio of regular TPU foams is usually less than 10-times, and they suffer from the significant post-shrinkage of the initial foams, mainly because of their low matrix modulus. HD-TPU presents a high matrix modulus and a rapid crystallization rate, facilitating the stabilization of the expanded cell structure and, hence, providing a good choice for obtaining a foam with an increased foam expansion ratio. Unfortunately, as indicated in the previous sections, HD-TPU foams with the maximum expansion ratio, i.e., 3.9-fold and 4.4-fold, were obtained by optimizing the saturation temperature and saturation time, which are even lower than in the 10-fold expansion of regular TPU foams [11]. Figure 11 demonstrated that the crystal domains with *T*_mx_ around 200 °C could exist at 15 MPa/170 °C for various saturation times, and Table 2 indicates that the *M*_w_ of HD-TPU foams are still high, i.e., 125.2–133.5 kg/mol. According to the generally accepted viewpoint, the presence of crystal domains and a reasonably high *M*_w_ could maintain “melt” strength and promote stable cell growth, further expanding the foam. However, the presence of coarsened cells and microcracks at saturation temperatures higher than 170 °C indicated that in addition to the “melt” strength requirement there could be another mechanism to dominate cell growth. Based on the degradation pathways indicated in Figure 12 and the cell structure evolution shown in Figure 10 and Figure 11, we believe that the degradation behavior of HD-TPU at high saturation temperatures could be the major factor to determine cell morphology.

**Figure 13:**
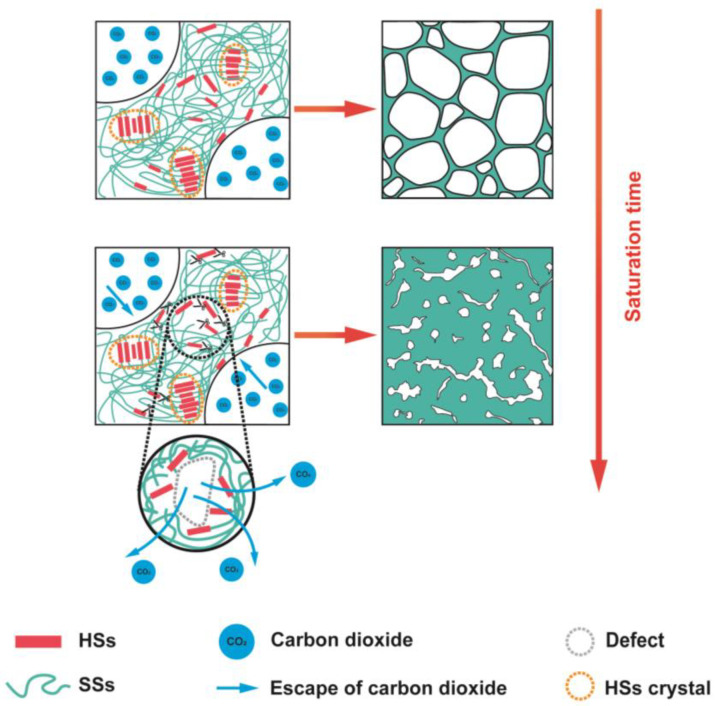
The mechanism of restricted expansion of HD-TPU foams.

Figure 13 shows the schematic diagram of the cell growth process at high saturation temperatures, which was inferred from the cell morphologies of HD-TPU foam obtained at 15 MPa/170 °C/5–90 min. The supercritical CO_2_ had high gas diffusivity and it could diffuse into polymer matrix very quickly, while it took longer for the heat transfer to reach uniform temperature inside the sample. The crystal-enhanced “melt” strength stabilized nucleated bubbles and promoted cell growth and foam expansion at the short saturation time of 5–15 min, as indicated in Figure 10. As time went on, significant thermal degradation occurred within the HD-TPU polymer matrix, some polymer chains broke down, and even some volatile molecules were generated, resulting in the formation of lots of defects within the HD-TPU matrix. During the foaming, these defects within the polymer matrix or cell wall could act as the gas escape channel, which enhanced gas diffusion from the growing bubbles, leading to the generation of coalesced cells and microcracks and the reduction in the foam expansion ratio. At the extended saturation time at 170 °C or high saturation temperature of 180 °C, the degradation degree was increased, and the formation of more defects in the HD-TPU matrix would inevitably accelerate the escape of supercritical fluid, resulting in more serious cell coalescence.

The occurrence of thermo-oxidative degradation is very common for polymers during high-temperature processing; however, very few studies have discussed the effect of degradation on cell growth. The possible reasons are that either the foaming temperature is much lower than the polymer degradation temperature or the polymer degradation degree at the foaming temperature is low. As indicated in Table 2 and Figure 10, the *M*_w_ reduction of about 1.9–12.6% did not spoil the foaming behavior of HD-TPU, whereas the HD-TPU foams obtained at 15 MPa/170 °C/5–15 min exhibited excellent cell morphology and low foam density. Once the *M*_w_ reduction reached 20.0–26.4%, the degradation tended to significantly destroy the cell morphologies of the HD-TPU foams. We speculated that the slight degradation might only induce a small number of defects or small-sized defects in the polymer matrix, which might not obviously affect cell growth. However, when more defects were formed within the polymer matrix at the increased degradation degree, the cell growth could be interrupted by rapid gas escape from these defect channels.

### 2.6. Hardness of HD-TPU Foams

**Figure 14:**
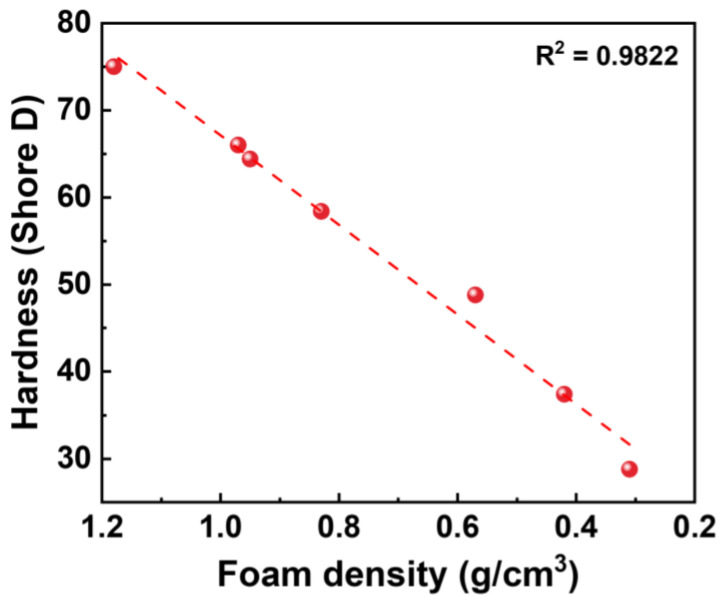
The relationship between foam density and hardness. The red dotted line represents the fitting result.

Hardness reflects the ability of materials to resist external forces and is an important indicator for many applications. It could be seen from Figure 14 that the introduction of cell structure reduced the hardness of HD-TPU foam, and its value decreased linearly with the decrease in foam density. This result is consistent with the variations in other elastomer foams [57]. Thanks to the high hardness of the unfoamed sample itself, the hardness of the foamed sample with a density of 0.31 g/cm^3^ is 29D. This shows that the use of high-hardness raw materials is an effective strategy to achieve the balance between low density and high hardness in TPU foam.

## 3. Experimental Section

### 3.1. Materials

HD-TPU pellets with Shore D hardness of 75D were purchased from Lubrizol Specialty Chemicals Manufacturing (Shanghai) Co., Ltd., with a melt index of 38.1 g/10 min (220 °C, 5 kg). The density of HD-TPU was 1.18 g/cm^3^. According to the NMR result, the HS was made of 4,4′-methylene diphenyl diisocyanate (MDI) and butanediol (BDO), the SS was polytetrahydrofuran (PTMG) with a molar mass of M_n_~1000 g/mol (Figure S2). CO_2_ with a purity of 99.9%, used as the physical blowing agent, was purchased from Guangzhou Guangqi Gas Corporation.

### 3.2. Sample Preparation

**Figure 15:**
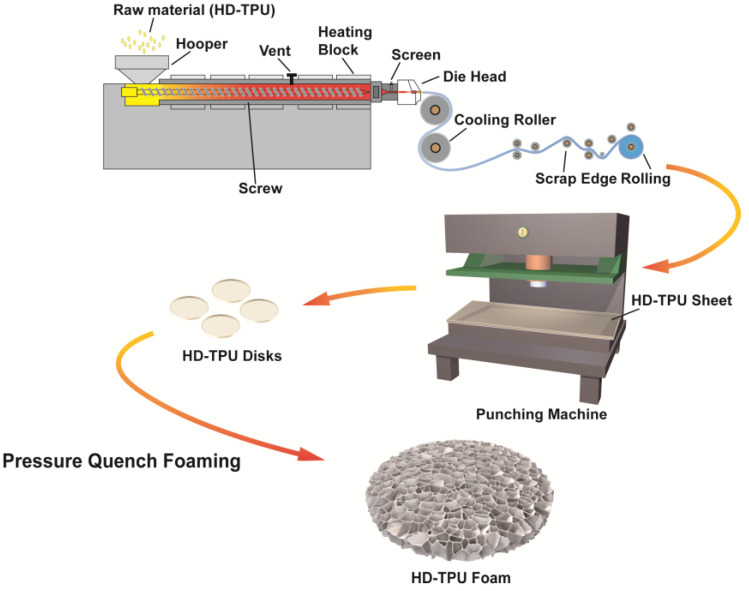
Schematic diagram of the preparation process of HD-TPU foams.

Sample preparation process is illustrated in Figure 15. TPU sheets with a thickness of 1.5 mm were produced by a plastic film extruder. The processing temperatures were in the range of 190–215 °C. HD-TPU disks with a diameter of 8 cm were obtained by a punching machine, and were used for foaming. HD-TPU foams were prepared by a pressure-quenching foaming process. Briefly, the HD-TPU disks were placed inside the high-temperature chamber and then the chamber was closed by hydraulic ram. Then, CO_2_ was fed into the chamber by a high-pressure syringe pump. After being saturated for various saturation times, the supercritical fluid was rapidly released in 5 s by a solenoid valve. Finally, the chamber was quickly opened through hydraulic ram and the foams were removed and cooled in the air.

### 3.3. Characterization

#### 3.3.1. Isothermal Annealing Analysis

DSC analysis was measured by DSC 250 (TA instruments, USA) at temperatures ranging from −50 °C to 250 °C. The materials were heated from −50 °C to various annealing temperatures (120, 140, 160, and 180 °C) and maintained for 30 min in order to conduct the isothermal annealing tests. The samples were then rapidly cooled to −50 °C and reheated to 250 °C to further understand how annealing affected the HSs ordering.

#### 3.3.2. FTIR Analysis

A Bruker INVENIO-R Fourier transform infrared spectrometer was used to measure the HD-TPU’s infrared spectra. With a diamond ATR probe, infrared spectral data were collected within the wavenumber range of 4000–400 cm^−1^ using a resolution of 4 cm^−1^ and 64 scans. Three HD-TPU disks from the same batch were subjected to time scans with the temperatures fixed at 150 °C, 170 °C, and 190 °C, respectively.

#### 3.3.3. DMA of HD-TPU

The dynamic mechanical properties of TPU were measured by a Dynamic Mechanical Analyzer (DMA 242D, Netzsch, Germany) under tension mode at a frequency of 1 Hz and an amplitude of 10 µm. The temperature was ramped from −100–180 °C at a heating rate of 5 °C/min. The dimensions of the samples were 20 mm × 6 mm × 1.5 mm.

#### 3.3.4. Thermal Properties of HD-TPU Foams

Thermal properties of HD-TPU foams were analyzed through DSC (DSC 250, TA Instruments, New Castle, DE, USA) within a temperature range of −50 °C to 250 °C and a heating rate of 10°C/min. Foam samples weighed at 3–4 mg were used for the test, and the normalized data were generated directly by TRIOS software provided by TA Instruments.

#### 3.3.5. Cellular Morphology

The density (*ρ*_f_) of HD-TPU foams was determined by a densitometer (DA-300 M, DahoMeter, China).

A scanning electron microscope (SEM) was used to analyze the cellular structure (COXEM EM 30AX Plus). The cell sizes of HD-TPU foams were determined using the Nano Measurer software based on SEM images. Cell density (*N*_0_) was calculated by the following equation:

(1) $$N_{0}={\left[\frac{n}{A}\right]}^{\frac{3}{2}}\phi$$

where *A* stands for the area of the SEM images (cm^2^), *n* denotes the number of cells. The expansion ratio ϕ of the HD-TPU foams was determined using the following equation:

(2) $$\phi =\frac{\rho_{s}}{\rho_{f}}$$

where *ρ*_s_ and *ρ*_f_ is the density of the unfoamed and foamed samples, respectively.

#### 3.3.6. Determination of Relative Molecular Weight and Dispersibility

The molecular weight and dispersibility of HD-TPU extruded sheet and foams were measured by GPC. Three PLgel gel chromatographic columns (10 m MIXED-B, 20 m MIXED-A, and 20 m MIXED-A) were used in the GPC system, which also included an Agilent 1260 Iso pump and a differential detector. Chromatographic grade N, N-dimethylformamide (DMF) contained 10 mmol/L lithium bromide was mobile phase. The flow rate of mobile phase was 1.0 mL/min, the column temperature was 50 °C. Polystyrene was used as the internal standard. The standard curve was determined by eight samples that *M*_p_ were 1140; 3050; 9820; 27,060; 67,600; 217,100; 696,000; and 2,189,000, respectively. Additionally, their PDI are 1.06, 1.04, 1.02, 1.02, 1.02, 1.04, 1.05, and 1.03, respectively. To completely dissolve the TPU, first 0.3 mg of HD-TPU and 1 mL of DMF were fed into a centrifuge tube, which was then heated in a 40 °C water bath for 15 min. Before injection, the solution was filtered with a 0.22 μm nylon filter.

#### 3.3.7. Hardness Characterization

In order to investigate the relationship between hardness and density of foams, the hardness of TPU foamed sheets were measured by a Shore D durometer (LX-A, HANDPI). The hardness reading was based on initial indentation in accordance with ASTM D2240-15. The given hardness values were calculated using the average of five separate spots on the foamed sheet surface.

## 4. Conclusions

In this study, the foaming behavior of HD-TPU with hardness of 75D was investigated, and a pressure-quenching foaming method using CO_2_ as a blowing agent was carried out. HD-TPU presented multiple melting behaviors with *T*_mx_ at 180.4–199.3 °C and a high matrix modulus up to 150 °C. A valley-shaped tendency of the HD-TPU foam density was observed with increasing saturation temperatures, and the foam with the maximum expansion ratio of 3.9-fold, cell size of 25.9 μm, and cell density of 7.8 × 10^8^ cells/cm^3^ was obtained at 18 MPa/170 °C/30 min due to the full cell growth with the decrease in the matrix modulus. With further increasing the saturation temperature, however, the density of the HD-TPU foam significantly reduced to 1.0 g/cm^3^ and coalesced cells occurred. DSC measurements indicated that crystal domains could exist at these foaming conditions, and they could act as physical crosslinking points to maintain “melt” strength and stabilize cell growth in theory. Our GPC test showed that obvious degradation occurred, and lots of defects could be generated within the HD-TPU matrix during this process. A possible mechanism of cell growth at the degradation environment was proposed, and the rapid gas escape from the growing bubbles through the defect channels could be a main reason for the formation of coalesced cells, microcracks, and a high foam density. The introduction of a microcellular structure reduced the ability of the polymer foam matrix to resist external forces, which tended to linearly reduce the hardness of HD-TPU foams.

## Figures and Tables

**Table 1:** The values of multiple melting peaks at different annealing temperatures.

	*T*_m_/°C	*T*_m1_	*T*_m2_	*T*_m3_	*T*_m4_	*T*_m5_
*T*_a_/°C						
Unannealed		51.2	180.4	190.0	199.3	/
100		118.4	182.3	190.8	200.5	/
120		135.7	182.3	190.4	200.6	/
140		154.8	182.3	190.0	200.5	/
160		176.5	183.2	190.8	200.8	/
180		/	192.5	194.4	201.8	212.6

**Table 2:** The RMW and PDI of unfoamed and foamed samples.

Sampels	Saturation Temperature (°C)	Saturation Time (min)	*M*_n_ (kg/mol)	*M*_w_ (kg/mol)	PDI
Unfoamed	/	/	101.2	181.5	1.8
Foam	170	5	102.2	178.0	1.7
Foam	170	15	93.5	158.6	1.7
Foam (upper)	170	30	85.4	144.7	1.7
Foam (lower)	170	30	79.8	133.5	1.7
Foam	170	60	72.9	121.2	1.7
Foam	170	90	72.1	120.7	1.7
Foam	180	30	74.8	125.2	1.7

## Data Availability

Data will be made available on request.

## Supplementary Materials

The following supporting information can be downloaded at: https://www.mdpi.com/article/10.3390/molecules27248911/s1. Figure S1: Retention time curves of foams and unfoamed sample; Figure S2: ^1^H NMR spectra of 75D raw material.

## Sample Availability

Samples of the HD-TPU foams are available from the authors.
