# Supplementary material for: Supercritical Fluid Microcellular Foaming of High-Hardness TPU via a Pressure-Quenching Process: Restricted Foam Expansion Controlled by Matrix Modulus and Thermal Degradation
Source: Molecules. 2022 Dec 15;27(24):8911. doi: 10.3390/molecules27248911 (PMC9783504; doi:10.3390/molecules27248911)
Supplement: Supplementary file 1 [file molecules-27-08911-s001.zip › molecules-2082781-supplementary.pdf]

Supporting Information

Supercritical Fluid Microcellular Foaming of High-Hardness TPU via a Pressure-Quenching Process: Restricted foam Expansion Controlled by Matrix Modulus and Thermal Degradation

Bichi Chen [1,†], Junjie Jiang [1,2,3,†], Yaozong Li [1], Mengnan Zhou [1], Zelin Wang [1], Liang Wang [1] and Wentao Zhai [1,*]

1 School of Materials Science and Engineering, Sun Yat-sen University, Guangzhou 510275, China
2 Ningbo Key Lab of Polymer Materials, Ningbo Institute of Materials Technology and Engineering, Chinese Academy of Sciences, Ningbo 315201, China
3 University of Chinese Academy of Sciences, Beijing 100049, China
* Correspondence: zhaiwt3@mail.sysu.edu.cn; Tel./Fax: +86-020-8411-3428
† These authors contributed equally to this work.

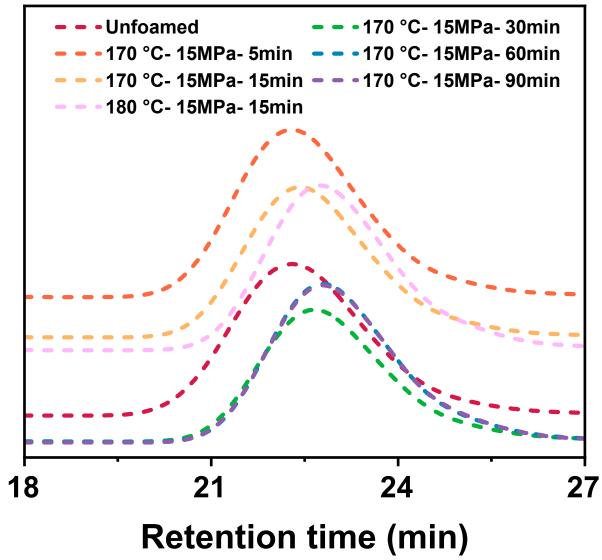

Figure S1. Retention time curves of foams and unfoamed sample. The saturation pressure were 15 MPa, and the saturation temperature was 170 °C or 180 °C with varying saturation time.

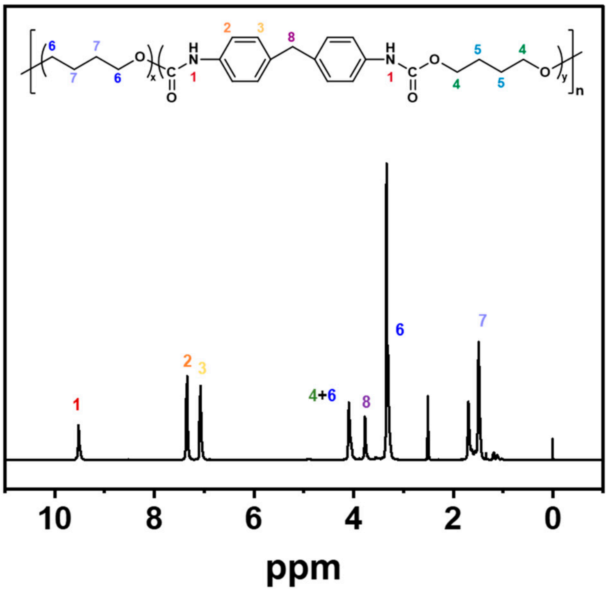

Figure S2. ^{1}H NMR spectra of 75D raw material.
